# Management of perforated diverticulitis with generalized peritonitis. A multidisciplinary review and position paper

**DOI:** 10.1007/s10151-020-02346-y

**Published:** 2020-11-05

**Authors:** R. Nascimbeni, A. Amato, R. Cirocchi, A. Serventi, A. Laghi, M. Bellini, G. Tellan, M. Zago, C. Scarpignato, G. A. Binda

**Affiliations:** 1 Department of Molecular and Translational Medicine, University of Brescia, Viale Europa 11, 25124 Brescia, Italy; 2 Unit of Coloproctology, Department of Surgery, Borea Hospital, Sanremo, Italy; 3 Department of Surgical and Medical Sciences, University of Perugia, Terni, Italy; 4 Department of Surgery, Galliano Hospital, Acqui Terme, Italy; 5 Department of Surgical-Medical Sciences and Translational Medicine, “Sapienza” University of Rome, Rome, Italy; 6 Gastrointestinal Unit, Department of Translational Research and New Technologies in Medicine and Surgery, University of Pisa, Pisa, Italy; 7 Department of Internal, Anesthesiological and Cardiovascular Clinical Sciences, “Sapienza” University of Rome, Rome, Italy; 8 Department of Robotic and Emergency Surgery, Manzoni Hospital, ASST Lecco, Lecco, Italy; 9 Department of Health Sciences, United Campus of Malta, Msida, Malta; 10 Faculty of Medicine, Chinese University of Hong Kong, ShaTin, Hong Kong; 11 General Surgery, Biomedical Institute, Genoa, Italy

**Keywords:** Diverticular disease, Acute diverticulitis, Diffuse peritonitis, Septic shock

## Abstract

Perforated diverticulitis is an emergent clinical condition and its management is challenging and still debated. The aim of this position paper was to critically review the available evidence on the management of perforated diverticulitis and generalized peritonitis in order to provide evidence-based suggestions for a management strategy. Four Italian scientific societies (SICCR, SICUT, SIRM, AIGO), selected experts who identified 5 clinically relevant topics in the management of perforated diverticulitis with generalized peritonitis that would benefit from a multidisciplinary review. The following 5 issues were tackled: 1) Criteria to decide between conservative and surgical treatment in case of perforated diverticulitis with peritonitis; 2) Criteria or scoring system to choose the most appropriate surgical option when diffuse peritonitis is confirmed 3); The appropriate surgical procedure in hemodynamically stable or stabilized patients with diffuse peritonitis; 4) The appropriate surgical procedure for patients with generalized peritonitis and septic shock and 5) Optimal medical therapy in patients with generalized peritonitis from diverticular perforation before and after surgery. In perforated diverticulitis surgery is indicated in case of diffuse peritonitis or failure of conservative management and the decision to operate is not based on the presence of extraluminal air. If diffuse peritonitis is confirmed the choice of surgical technique is based on intraoperative findings and the presence or risk of severe septic shock. Further prognostic factors to consider are physiological derangement, age, comorbidities, and immune status. In hemodynamically stable patients, emergency laparoscopy has benefits over open surgery. Options include resection and anastomosis, Hartmann’s procedure or laparoscopic lavage. In generalized peritonitis with septic shock, an open surgical approach is preferred. Non-restorative resection and/or damage control surgery appear to be the only viable options, depending on the severity of hemodynamic instability. Multidisciplinary medical management should be applied with the main aims of controlling infection, relieving postoperative pain and preventing and/or treating postoperative ileus. In conclusion, the complexity and diversity of patients with diverticular perforation and diffuse peritonitis requires a personalized strategy, involving a thorough classification of physiological derangement, staging of intra-abdominal infection and choice of the most appropriate surgical procedure.

## Introduction

In the past few decades, the complicated diverticular disease has been an increasing burden on healthcare systems as its incidence has steadily risen in Western countries [1–3] including Italy [4]. Moreover, the rate of peritonitis-related mortality remains high [5].

The treatment of complicated intra-abdominal infections, including perforated diverticulitis with peritonitis, has been changing recently, mainly due to the evolution of medical/intensive care and the development of multidrug-resistant bacteria.

Relevant aspects of the management of perforated diverticulitis remain controversial for several reasons: high-quality data are lacking, and the main guide for therapeutic choices, the Hinchey classification [6, 7], is proving insufficient in the age of personalized surgery, with indications and outcomes being substantially influenced by surgical-teams’ specialization. At the same time, physiological patterns are becoming critical in decision-making, challenging surgical approaches with different clinical scenarios.

To review the current evidence and to rationalize the management of perforated diverticulitis with peritonitis, four Italian scientific societies (the Italian Society of Colon & Rectal Surgery [SICCR], the Italian Society of Emergency & Trauma Surgery [SICUT], the Italian Society of Hospital Gastroenterologists & Digestive Endoscopy [AIGO], and the Italian Society of Medical & Interventional Radiology [SIRM]) endorsed a project involving professionals with different clinical expertise, including surgeons, gastroenterologists, radiologists and intensivists (see “Methodological notes”).

The aim of this position paper was to critically review the available evidence on perforated diverticulitis and generalized peritonitis, focusing on relevant key points with the main goal of developing a comprehensive decision-making pathway for the management of patients with this condition.

## Methodological notes

The four scientific societies (SICCR, SICUT, SIRM, AIGO), identified experts amongst their members to set-up the Scientific Committee, which defined the methodology to be followed in the preparation of the position paper. The methodology adopted to process the recommendations consisted of four steps. In a Technical Committee meeting, held at the beginning of 2019, the Scientific Committee identified 5 clinically relevant issues in the management of perforated diverticulitis with generalized peritonitis that would benefit from a multidisciplinary review.

Each selected topic was assigned to two experts, who carried out an independent systematic search of the relevant literature using Medline/PubMed, Embase, and the Cochrane databases. Search outputs were distilled, paying more attention to systematic reviews and meta-analyses (where available).

For each topic, a draft was prepared and circulated amongst all the members of the Scientific Committee. Each expert then provided his/her input. Following preparation of the revised draft, each topic was addressed to the Core Writing Group, who prepared the first draft of the full manuscript, which was examined during another Technical Committee meeting in October 2019. During the meeting, each single topic was thoroughly discussed and each statement concerning the summary of current evidence refined with regard to both content and wording.

The Core Writing Group then incorporated all the suggestions raised during the digital meeting and prepared the final draft. In doing so, an updated literature search was performed and the most recent evidence included. Any changes resulting from comments received by the experts were made on the basis of scientific and editorial merit to produce the final version of the position paper, which was then sent to all the members for review and approval.

## Key Point 1

What criteria should be used to decide between conservative and surgical treatment in case of perforated diverticulitis with peritonitis?

The therapeutic choice should be personalized according to the severity of peritonitis and sepsis. Further prognostic factors to consider are physiological derangement, age, comorbidity, and immunocompetence.

Surgery is indicated in case of diffuse peritonitis or failure of conservative management. Extra-luminal air only is not an indication for emergency surgery but close monitoring is mandatory to detect treatment failure early.

### Considerations

Generalized peritonitis is the main indication for emergency surgery for perforated diverticulitis [8]. Nonetheless, several situations, are now approached non-operatively and eventually converted to elective surgery [9].

The accuracy of the initial diagnosis of generalised peritonitis is of paramount importance, but clinical evaluations rely on empirical rationale [10] as the evidence is poor. They should include physical signs and laboratory measures, together with parameters of hemodynamic instability, physiological derangement and organ dysfunction. Classical physical signs and laboratory markers have low accuracy in identification and staging of diffuse peritonitis [11, 12]; thus, the initial evaluation must incorporate a computed tomography (CT) scan and sepsis risk assessment.

Even though CT scan is considered the “gold standard” in diagnosing and staging perforated diverticulitis, the results are not always reliable [13]. Confirming previous observations [14], Gielens et al. [15], in a retrospective series of 75 patients, have shown that the combination of extra-luminal air with free fluid is the CT parameter most strongly associated with diverticular perforation and generalized peritonitis (positive predictive value: 80%). In the same study, however, the authors reported 42% of Hinchey 3 cases under-staged as Hinchey 1 or 2. Moreover, the CT scan is hardly able to differentiate between Hinchey 3 and 4 peritonitis (unless fecal material can be clearly distinguished) with a potential risk of under-staging [16]. In doubtful cases, a diagnostic ultrasound (US)-guided peritoneal aspiration might be helpful [17].

As regards extra-luminal air, a recent review [18] of 8 studies including 251 patients with isolated pericolic air showed an emergency surgery rate of 6%, demonstrating that conservative therapy is appropriate in such cases. Data on distant extra-luminal air are more controversial and scarce, although some retrospective studies have shown that a proportion ranging from one to two-thirds of those who have this condition are effectively treated with aggressive medical management [19–21].

Again, the association of distant free air with free fluid is the most commonly reported predictor of early failure of the non-operative approach requiring urgent surgery in more than 80% of cases [22, 23].

Early recognition of frail patients at risk of sepsis or failure of a non-operative approach is mandatory so that a lower threshold for timely surgical control of the source of infection can be set. Risk of sepsis may be defined according to the Sequential Organ Failure Assessment (SOFA) score (formerly known as the Sepsis-related Organ Failure Assessment score), or by simplified, but less sensitive, scores such as the quick SOFA (qSOFA) [24]. Assessment of septic shock is detailed in the next key point.

Further prognosticators of poor outcome in abdominal infections are advanced age, severe chronic cardiovascular and kidney disease, coexistent malignancy and immunodeficiency [25, 26]. In perforated diverticulitis, however, high American Society of Anesthesiologists (ASA) score [20], severity of sepsis [23] and intraabdominal parameters [19–23] seem more relevant predictors of failure of conservative management. All these factors must be carefully weighed to maintain a low threshold for surgery or conversion in the most frail patients.

## Key Point 2

What criteria or scoring system should be applied to choose the most appropriate surgical option when diffuse peritonitis is confirmed?

**Fig. 1:**
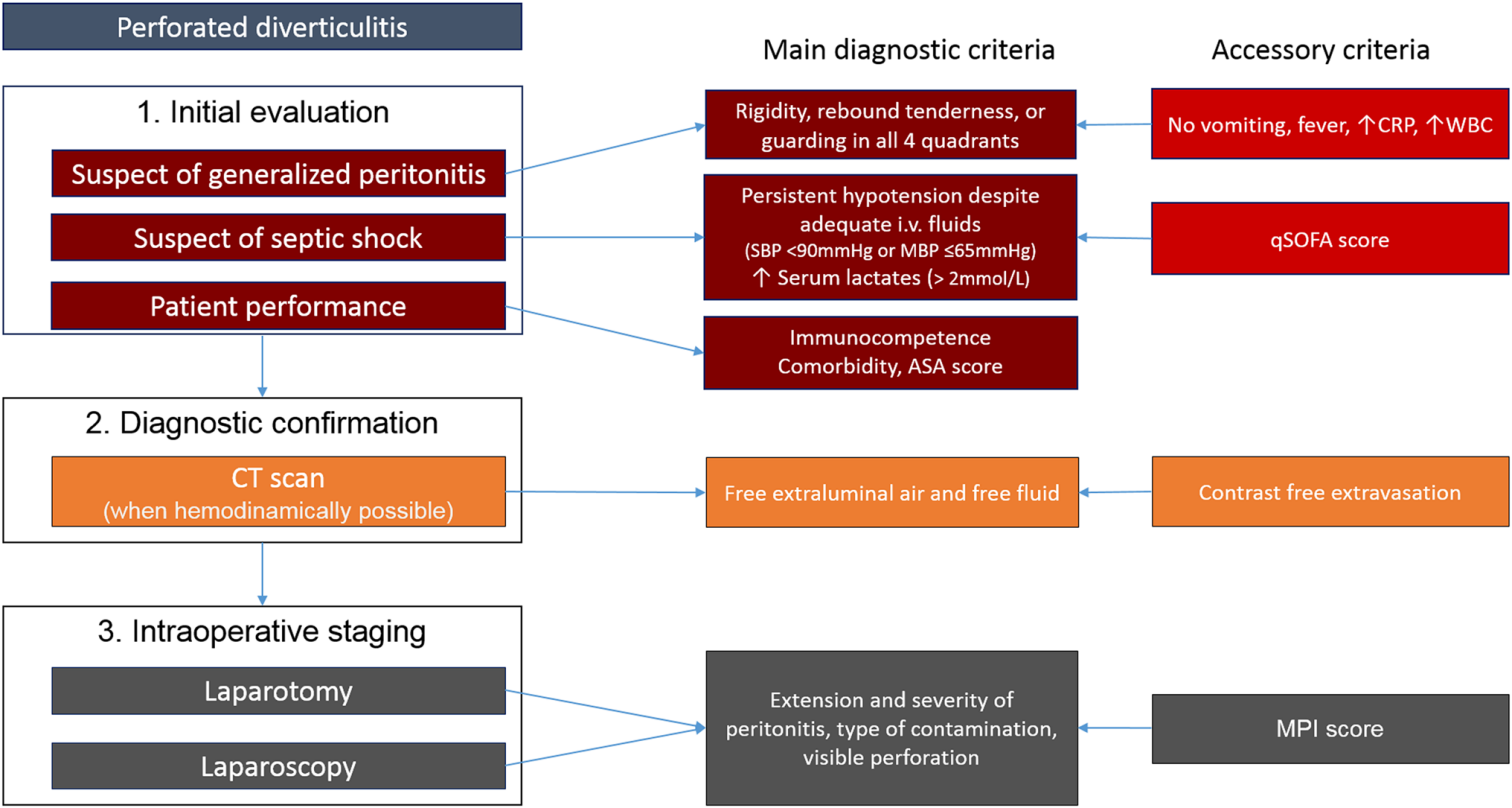
Multi-Step Decisional Approach for patients with diverticular perforation and diffuse peritonitis. *SBP* systolic blood pressure, *MBP* mean blood pressure, *qSOFA* quick Sequential [Sepsis-related] Organ Failure Assessment; *MPI* Mannheim Peritonitis Index

In case of confirmed diffuse peritonitis, there is no single staging or scoring system that alone can suggest the most appropriate surgical procedure. A stepwise decision-making approach must be applied weighing risk of septic shock, patient conditions, and the intraoperative abdominal findings (Fig. 1).

### Considerations

Up to now, the Hinchey classification has been guiding surgical choices according to the type of peritoneal contamination [3]. The evolution of knowledge and the increased variety of surgical options mandate a more articulated and tailored approach. Previous scoring systems [27–29] had limited spread in clinical practice.

When generalized peritonitis is diagnosed and an emergency surgical procedure is required, we suggest *a stepwise decisional approach.*

*Step 1* is an early assessment of septic shock. According to the SEPSIS-3 definition [24], septic shock is a “subset of sepsis in which underlying circulatory and cellular/metabolic abnormalities are profound enough to substantially increase mortality. Patients with septic shock can be identified with a clinical construct of sepsis with persisting hypotension requiring vasopressors to maintain mean arterial pressure ≥ 65 mm Hg and having a serum lactate level > 2 mmol/L despite adequate volume resuscitation”. In a trauma setting, the Advanced Trauma Life Support (ATLS) manual [30] recommends that “patient’s response to initial fluid resuscitation is the key to determining subsequent therapy” and the emergency team modifies the treatment strategy accordingly.

According to ATLS, response to initial fluid administration can be divided into three patterns:

- *Rapid responder group* patients respond to the initial fluid load restoring hemodynamic normality,
- *Transient responder group* patients respond initially, but quickly hypotension and tachycardia return,
- *Not responder group* patients remain unstable in spite of appropriate fluids.

In generalized peritonitis and septic shock, the definition of hemodynamic responsiveness versus unresponsiveness may offer guidance for timing (urgent versus emergent), type of primary approach (laparoscopic versus open-resuscitative), and choice of surgical procedure (see “Key-Point 4”). Depending on clinical settings, different screening tools, such as qSOFA, may also be added, bearing in mind their low sensitivity.

*Step 2* defines the overall fitness to surgery, investigating general performance and comorbidity. Severe comorbidities, immune deficits, advanced age and physiological status are longtime predictors of poor outcome for non-resective [31, 32] or restorative [33–35] procedures. Accordingly, they must be incorporated in preoperative decision-making of procedure selection in stable patients (see “Key-Point 3”), possibly by means of score systems [36–38].

*Step 3* is the intraoperative assessment of peritonitis severity and of perforation features. No available classification offers evidence-based guidance for procedure selection. Diffuse fecal contamination in the Hinchey classification is predictive of postoperative admission to the intensive care unit (ICU) and mortality [25, 29, 39, 40] but the choice of a non-restorative procedure, in this case, is made on empirical grounds. Among the newer and more detailed prognostic tools, the Peritonitis Severity Score [19] and the Mannheim Peritonitis Index (MPI) have been validated for scoring left colonic peritonitis according to mortality risk [41] but include few intraoperative parameters. However, in the absence of specifically validated systems, at present time the MPI is a simple score with an acceptable balanced evaluation of general and local derangements including time from the onset of the disease, diffusion and type of exudate.

Further intraabdominal visual parameters that have a significant association with procedure-specific adverse events are emerging [40]. For example, a visible free perforation [32] reduces the rate of sepsis control after laparoscopic peritoneal lavage (LPL).

## Key Point 3

What type of surgical procedure and approach is appropriate in hemodynamically normal or normalized patients with diffuse peritonitis?

**Fig. 2:**
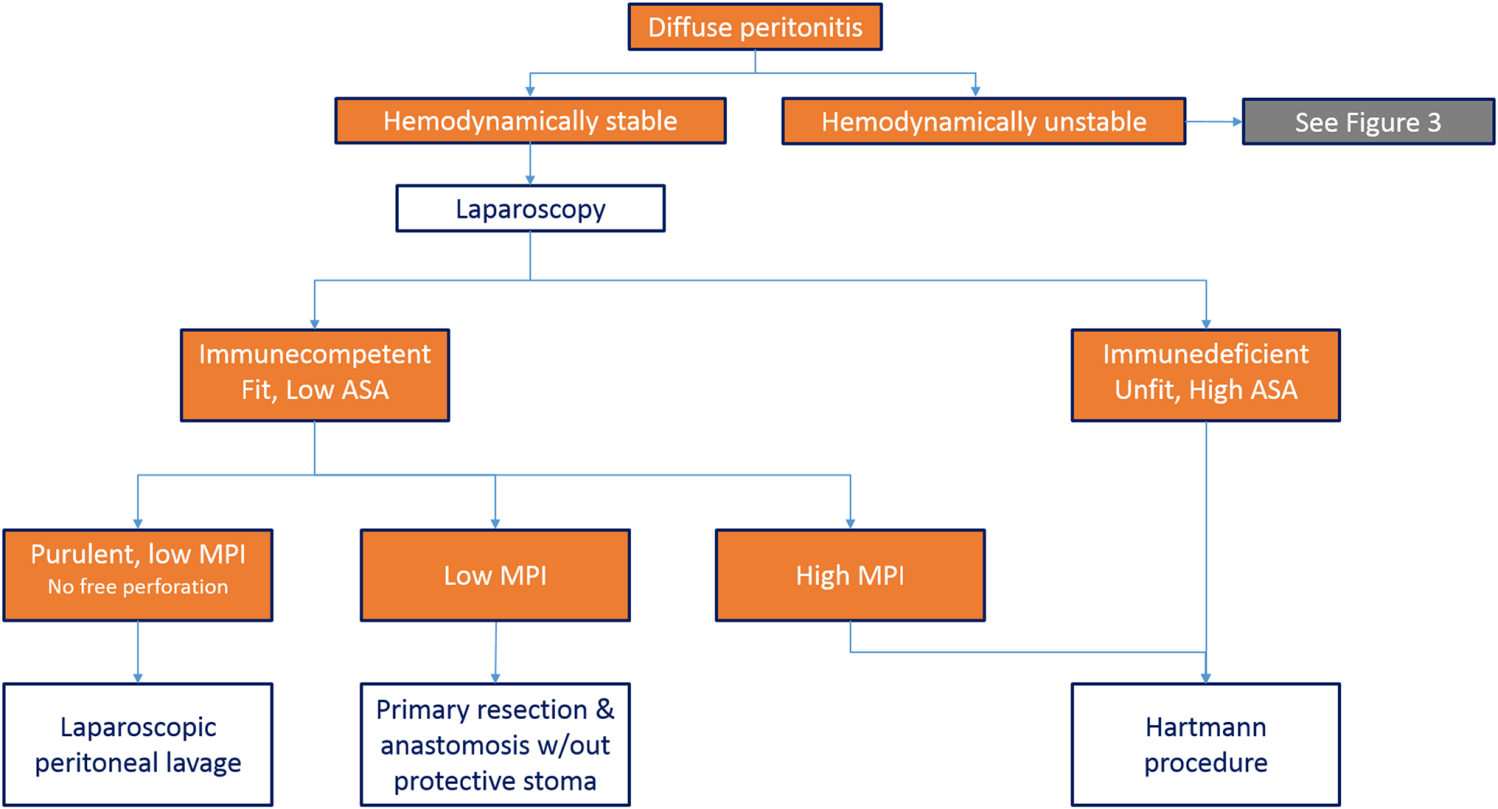
Treatment algorithm for hemodinamically stable patients. *MPI* Mannheim Peritonitis Index

- In hemodynamically stable patients, confirmation and staging of diffuse peritonitis may be obtained by laparoscopy (Fig. 2). In centers with adequate expertise, selected cases may be handled by emergency laparoscopic procedures, either resective or non-resective.

### Considerations

In stable patients with perforated diverticulitis and diffuse peritonitis, the initial laparoscopic approach has both diagnostic and therapeutic roles.

As stated previously, intra-abdominal vision makes it possible to confirm or rule out the CT diagnosis of generalised peritonitis, while allowing its staging. In selected cases (see “Key Point 3b”) LPL remains a feasible option when appropriate. Otherwise, even the resective procedure may be completed laparoscopically by surgeons and in centers with adequate expertise.

A few favourable outcomes of emergency laparoscopic resection for perforated diverticulitis have emerged. A meta-analysis [42] of experimental/observational studies comparing laparoscopic and open sigmoidectomy in acute settings showed reduced morbidity after a laparoscopic approach, although differences between the two groups in the rate of Hartmann's procedure (HP) vs primary resection and anastomosis (PRA), operating time, reoperation rate and postoperative 30-day mortality were not detected. Caution is advised when considering these data since lack of hemodynamic data and reasons for operative approach suggests a potential selection bias. Lately, a large retrospective study concluded that postoperative morbidity and mortality after emergency laparoscopic sigmoid resection for diverticular perforation and purulent peritonitis has morbidity (including leakage rate) and mortality rates similar to those of elective sigmoidectomy [43]. Moreover, in stable patients with unfavorable risk assessment, laparoscopic HP might be an attractive option because the risk of incisional hernia is minimized and the adhesion formation is reduced, facilitating reversal [44].

However, emergency laparoscopic resection for large bowel perforation may be a challenging procedure and the quality of present evidence remains inadequate to suggest a liberal use of this approach. Accordingly, we recommend that only experienced laparoscopic surgeons in laparoscopic centres of excellence perform it.

b. LPL may be effective in the management of purulent peritonitis reducing the rate of ostomy in selected patients. Its non-selective use results in high rates of unresolved sepsis and unplanned surgery.

### Considerations

LPL was proposed in 1996 [45] with initial favorable results [46–48] encouraging its unselective use in perforated diverticulitis with peritonitis [49]. Three subsequent randomised controlled trials (RCTs) comparing LPL to sigmoidectomy obtained different results [50–52] Recent meta-analyses also reached different conclusions on the 30-day and 90-day reoperation risk, possibly due to methodological flaws [53–60].

Some findings are conclusive: resolution of sepsis and early morbidity and mortality rates after LPL are similar to those after sigmoidectomy, rates of abscesses and unplanned reoperations at index admission are higher after LPL, and total reoperation rate (including planned stoma closure), permanent stoma rate and costs [61–63] are lower after LPL than after resective surgery.

A large retrospective multicenter study analysed LPL outcomes utilizing a detailed database on patient and disease characteristics and surgical technique [32]. LPL emerged as an adequate treatment for stable patients with diffuse purulent peritonitis, with low ASA and MPI scores and no visible free perforation. According to this study, most free perforations were discovered after an extensive adhesiolysis conducted by some surgeons to search for infected collections; for this reason, adhesiolysis has to be avoided or kept to a minimum during LPL.

These data highlight the need for a prospective large multicenter study to confirm or improve the selection of patients to be treated with LPL. Recurrent symptoms and need for further surgery after LPL at two years compare well with Hartmann’s resection and subsequent closure [64, 65], but no studies have compared LPL with primary resection and anastomosis.

c. When LPL is judged inappropriate, primary resection and anastomosis with or without protective ostomy, and non-restorative resection are both adequate. In the absence of solid evidence, selection criteria should be based on general and local predictors of the risk of the unsafe anastomosis.

### Considerations

RCTs comparing PRA with HP found no significant difference in mortality and overall morbidity after the index procedure, but all studies were prematurely terminated due to slow patient recruitment or for safety reasons [66–68]. Likewise, meta-analyses of experimental/observational studies and RCTs reported similar rates of major complications and postoperative mortality both for Hinchey III and IV disease [58, 69–71].

More recently, the LADIES trial found no significant differences in short-term outcome both in Hinchey III and IV patients, although this study also ended prematurely confirming the trial’s vulnerability to discontinuation in the emergency setting, a feature that potentially leads to an overestimation of treatment benefits [72].

Studies have shown that the permanent stoma rate is higher after HP whereas the likelihood of stoma reversal is higher after PRA [58, 66, 70]. In the LADIES trial, 12-month stoma-free survival was significantly better after PRA with a significantly shorter median time to reversal [72]. Furthermore, reversal is associated with a significantly lower morbidity in PRA than after HP [58, 72, 73].

Although many surgeons are reluctant to consider a primary anastomosis in the acute setting and the frequency of HP has remained unchanged over the last years [74–77], these results suggest that PRA is the optimal resective procedure in physiologically normal and immunocompetent patients with either purulent or fecal peritonitis. However, there is no gold standard to determine the indication for a non-restorative versus a restorative procedure, and as stated in previous key points, patient-related factors and severity of intraoperative findings, as well as surgeon expertise, should be the main criteria for tailoring surgical decision-making in this setting. A diverting stoma does not prevent clinical anastomotic leakage after PRA but can mitigate its detrimental effects [78]. In most RCTs a diverting stoma was performed according to the study design [66, 68] whilst it was left to the surgeon’s discretion in the LADIES trial [72]. In the latter, morbidity was not significantly different between the patients with (27%) vs without (73%) a stoma but a selection bias cannot be ruled out. In a retrospective analysis of a series of patients who had laparoscopic sigmoidectomy without diversion, Dreifuss et al. found a leakage rate of 5.7% and 5.4% in the elective and emergent setting, respectively [43]. No recommendations can be made concerning intraoperative colonic lavage before PRA in acute diverticulitis as only small not randomized series are reported in the literature [79–81].

## Key Point 4

What surgical option is adequate for patients with generalized peritonitis and septic shock?

**Fig. 3:**
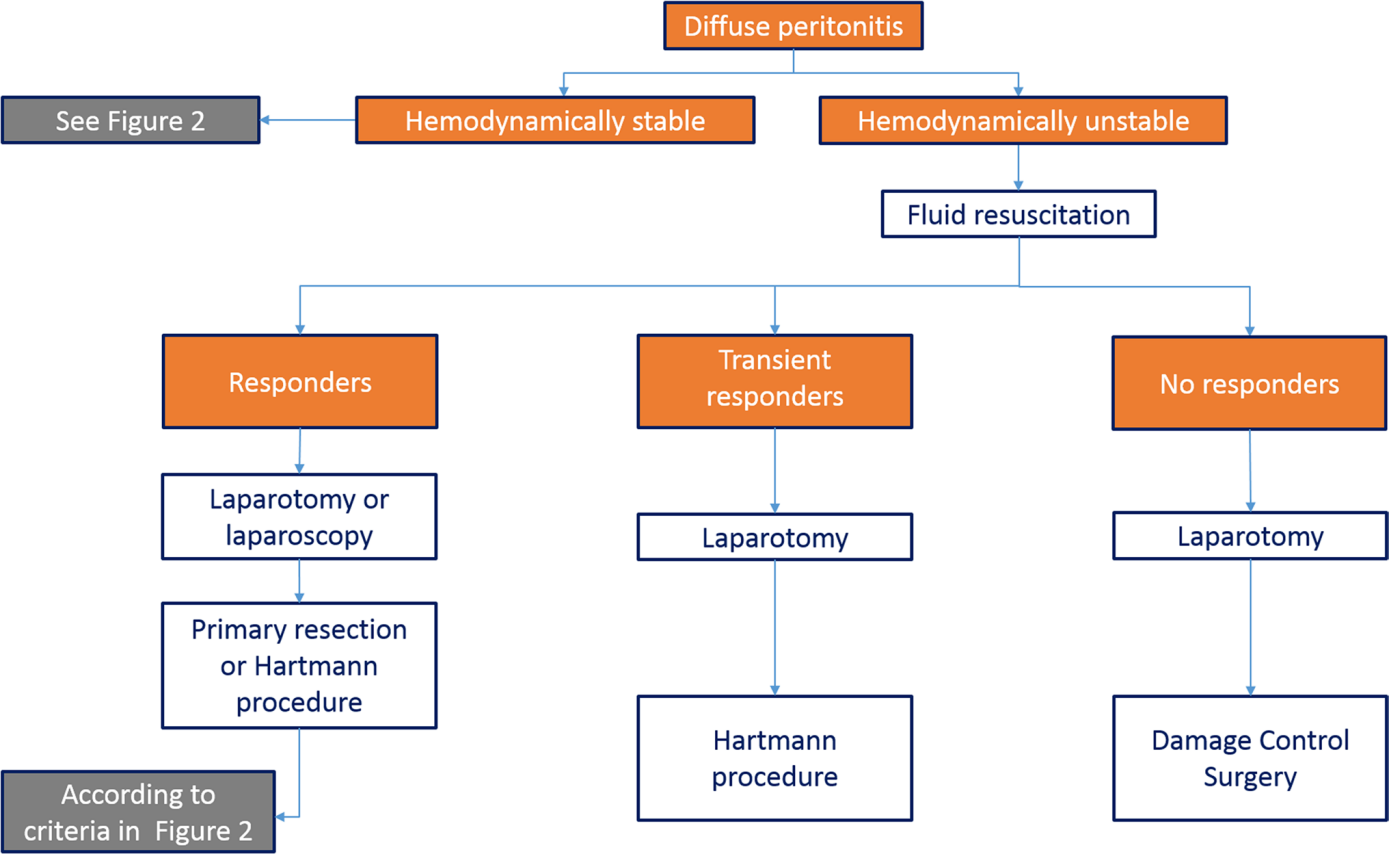
Treatment algorithm for hemodinamically unstable patients

In generalized peritonitis associated with septic shock, an open surgical approach is mandatory. Non-restorative resection and/or damage control surgery appear to be the only viable procedures, to be applied according to the severity of hemodynamic instability (Fig. 3).

### Considerations

In septic shock from secondary generalized peritonitis, the primary goals are controlling the source of abdominal infection and restoring organ perfusion and tissue oxygenation. As in traumatic shock [30], surgical treatment must be modulated according to hemodynamic conditions.

In the *Rapid Responder group*, after the restoration of hemodynamic stability, surgery is carried out immediately and the choices among approaches and procedures are made according to principles and criteria mentioned in key point 3.

In the *Transient Responder group*, the temporary return of hemodynamic instability restricts surgical options to HP or to damage control surgery (DCS), which are chosen based on the evolution of the physiological derangement and secondarily on intra-abdominal severity assessment.

In *Not-Responder group*, DCS is the most rationale immediate approach, due to the extreme exhaustion of the patient’s physiological reserves.

DCS is a three-stage approach typically involving:

- *Stage I* an abbreviated initial operative procedure with temporary abdominal closure;
- *Stage II* ICU resuscitation and management of physiological derangement;
- *Stage III* definitive treatment and closure of the abdominal wall, as soon as physiology is restored.

In major trauma, where it was initially described, the main goal of DCS is the prevention of the 'lethal triad': acidosis, hypothermia, and coagulopathy [82]. In secondary peritonitis, DCS aims at controlling the source of infection in the fastest and least invasive way, leaving completion surgery for a subsequent phase after adequate patient stabilization in the ICU [83].

In the subgroup of patients, in which the response to shock is strongly conditioned by the use of sympathomimetic amines, the synergy of behavior, intent, and timing among specialists is crucial. Monitoring the efficacy and side effects of vasoactive and inotropic pharmacologic support is also of paramount importance [84]. In fact, amines, especially norepinephrine, may lead to perfusion defects due to vasoconstriction of microcirculation, especially in the splanchnic circulation. On the other hand, failure to maintain an adequate cardiovascular balance during sympathomimetic support is associated with a higher risk of multi-organ insufficiency syndrome and unfavorable prognosis [85].

## Key Point 5

How should medical therapy support patients with generalized peritonitis from diverticular perforation before and after surgery?

Careful multidisciplinary medical management should be adopted with the main aims of:

- Controlling infection
- Relieving postoperative pain
- Preventing and/or treating postoperative ileus (POI)

### Considerations

A key component of the first-line management of the septic patient with peritonitis is the administration of intravenous antimicrobial therapy, before and after surgery. Antimicrobial therapy plays a pivotal role in the management of intra-abdominal infections, preventing multiple organ dysfunction caused by the ongoing peritoneal triggers. Indeed, an insufficient or otherwise inadequate antimicrobial regimen is one of the variables more strongly associated with unfavorable outcomes in critically ill patients [86].

Broad-spectrum antimicrobial therapy, including coverage against anaerobic microorganisms, is needed [87]. The empirically designed antimicrobial regimen (be it single or combined) is based on the underlying severity of infection, the pathogens presumed to be involved, and the risk factors indicative of major resistance patterns. Subsequent modification of the initial regimen may be possible later, on the basis of culture results (if available) and the patient’s clinical status. Indeed, the pathophysiological changes occurring during sepsis as well as the patient’s immunological status may significantly affect drug disposition in critically ill patients [88].

It has not yet been established which antimicrobial regimen is the best. A Cochrane systematic review [89] included 40 studies with 5094 patients and compared 16 different antibiotic regimens. All antibiotics showed equivocal comparability in terms of clinical success. As a consequence, no specific recommendations can be made for first-line treatment. Other factors such as local guidelines and preferences, ease of administration, costs and availability must, therefore, be taken into consideration when deciding on the antibiotic regimen. The most widely recommended dosing regimens (and their modifications according to renal function) are discussed in detail in the WSES position paper [90].

The spread of antimicrobial resistance is one of the leading public health problems worldwide and has been accelerated by the overuse and misuse of antimicrobial drugs [91]. According to guidelines, the diagnostic algorithm for a range of bacterial infections comprises a measurement of serum procalcitonin to identify sepsis [92] and to guide antimicrobial therapy [93].

Poorly controlled acute postoperative pain is associated with increased morbidity, functional and quality-of-life impairment, delayed recovery time, prolonged duration of analgesic drug use, and higher healthcare costs.

Conventional opioids remain the standard of care for the management of acute postoperative pain; however, the risk of opioid-related adverse events can limit optimal dosing, leading to poor pain control. To this end, multimodal analgesia should be applied [94] to improve analgesic effect, to reduce the doses of any single agent, and to minimize risks of untoward effects [95, 96].

Intravenous patient-controlled analgesia (PCA) is recommended over healthcare provider-initiated intermittent bolus dosing of opioids since current evidence shows greater effectiveness and patient satisfaction. Intravenous boluses of opioids might be considered during the first several hours after surgery for faster pain relief and analgesic titration [97]. Paracetamol, pro-paracetamol and nonsteroidal anti-inflammatory drugs (NSAIDs) are recommended as components of multimodal analgesia. A systematic review found that paracetamol and COX-2 selective and non-selective agents, added to PCA, significantly reduced morphine consumption [97].

Alarmingly, 4 recent meta-analyses [98–101] found that NSAID use is significantly associated with a higher risk of anastomotic leakage. This effect seems to be molecule-specific (diclofenac is associated with the highest risk) [101] and class-specific (being non-significant with COX-2 selective agents) [100, 101]. Furthermore, the risk varies with the duration of the treatment, and it is higher after 3 or more days of NSAID treatment than after 1 or 2 days only [102]. Although the balance of benefit versus risk (analgesic effect/risk of anastomotic disruption) may be acceptable in elective surgery, perioperative NSAIDs (especially given for more than 48 h) in emergent colorectal surgery should be evaluated carefully and on an individual basis, taking into account the presence of risk factors for anastomotic leakage (i.e. advanced age, malnutrition, severe comorbidities, intraoperative difficulties and complications).

Amongst regional anesthetic techniques, epidural analgesia remains the golden standard for postoperative pain control in patients undergoing open abdominal surgery [102, 103], allowing for a faster recovery of gastrointestinal transit and reducing the length of hospital stay [104].

POI has a multifactorial etiology and can deeply affect the patient’s recovery, lengthening hospital stay and increasing costs [105, 106]. The incidence of POI after colorectal surgery ranges from 15 to 19% [107, 108] and 60% of patients have a severe clinical presentation [82]. Intraoperative blood loss, administration of any intravenous opioids in the first 48 h, postoperative epidural analgesia and non-compliance with intraoperative fluid management protocols are predictors of POI [109].

Although many different options (nasogastric tube, fluid restriction, colloid versus crystalloid combinations, early feeding, prokinetics) are available, management of POI is still a matter of debate, albeit rapidly evolving [110].

Chewing gum as a form of sham feeding is an inexpensive and well-tolerated means of promoting gastrointestinal motility following major abdominal surgery. A recent meta-analysis [111] found that the incidence of POI is significantly reduced in patients using chewing gum.

Till now the use of prokinetics in the treatment of POI has been disappointing even if prucalopride, a highly selective 5-HT4-receptor agonist, can shorten POI and improve recovery time, as well as reduce intestinal inflammation [110].

A meta-analysis [112], evaluating both the efficacy and safety of the currently available drugs for prevention of POI, concluded that, despite study inconsistency (due to heterogeneity of endpoints) and lack of head-to-head studies, there is enough evidence to recommend the use of alvimopan, a peripherally-acting mu-opioid receptor antagonists (PAMORA) in major gastrointestinal surgery. Two further meta-analyses [113, 114] confirmed that- after *open* abdominal surgery this PAMORA can accelerate recovery of gastrointestinal function, shorten the length of hospital stay, and reduce POI-related morbidity without compromising opioid analgesia, resulting in a cost-saving approach [115].

## Limitations and controversies

The lack and poor quality of scientific data for relevant aspects of the management of patients with diverticular perforation and generalized peritonitis are the most obvious limitation to the construction of a comprehensive evidence-based strategy.

Feasibility of randomized studies in this surgical scenario remains questionable [116], as demonstrated by the premature interruption of the 4 trials comparing PRA and HP [66–68, 72] and by the conflicting results of the 3 trials comparing resection techniques and LPL [50–52]. Possibly, a well-designed and powered prospective cohort trial could be a more realistic and efficient alternative in these settings.

In other instances, the limitation is the near-total absence of adequate studies. The diagnostic and staging framework of diverticular perforation with peritonitis is a good example of that, with the accuracy of clinical and radiological markers rarely investigated so far.

Another constraint on the applicability of general recommendations for the surgical management of perforated diverticulitis with peritonitis is the different attitude of colorectal surgeons and non-colorectal specialists, who perform the vast majority of procedures for complicated diverticulitis [117, 118] but with significantly lower rates of PRA [117, 118] and LPL [118]. A recent US nationwide analysis on 2729 emergency surgeries found only 7.6% were PRA [119]. The surgeon’s expertise and specialization is an important factor in the decision-making process and leads to disparities not only in the choice of the procedure but also procedure-specific outcomes, such as ostomy rate, morbidity and even mortality [117, 118, 120].

## Future perspectives

Among the topics for future investigation, we believe that five deserve the highest priority.

First, the role and accuracy of physical, laboratory and radiological parameters for diagnosing and staging diverticular peritonitis need better definition with adjustment benchmarks according to the general and physiological status of patients. Validated criteria with cut-offs will allow better triage between medical and surgical approach.

Second, intraoperative findings should offer additional guidance to choose the most effective and safe procedure. However, the exact meaning of intra-abdominal visual features, including purulent or fecal contamination, awaits further investigation and incorporation into validated scoring tools.

Third, prospective studies are advisable to demonstrate the efficacy of damage control laparotomy in the most fragile patients with septic shock.

Fourth, the few available data on Enhanced Rehabilitation protocols after emergency colorectal surgery show that compliance may be lower than in the elective setting [121]. Accordingly, their feasibility and efficacy in patients undergoing surgery and eventually intensive care for diverticular peritonitis remain to be addressed.

Fifth, functional and patient-reported outcomes are mostly overlooked goals in emergency settings and need further dedicated insights.

## References

[1] Lamm R, Mathews SN, Yang J (2017). 20-year trends in the management of diverticulitis across New York state: an analysis of 265,724 patients. J Gastrointest Surg.

[2] Hong MKY, Skandarajah AR, Higgins RD, Faiz OD, Hayes IP (2017). International variation in emergency operation rates for acute diverticulitis: insights into healthcare value. World J Surg.

[3] Hupfeld L, Pommergaard HC, Burcharth J, Rosenberg J (2018). Emergency admissions for complicated colonic diverticulitis are increasing: a nationwide register-based cohort study. Int J Colorectal Dis.

[4] Amato A, Mataloni F, Bruzzone M (2020). Hospital admission for complicated diverticulitis is increasing in Italy, especially in younger patients: a national database study. Tech Coloproc.

[5] Coccolini F, Trevisan M, Montori G, et al (2017) Mortality Rate and Antibiotic Resistance in Complicated Diverticulitis: Report of 272 Consecutive Patients Worldwide: A Prospective Cohort Study. Surg Infect (Larchmt). 2017 Jul 21 10.1089/sur.2016.283 28731836

[6] Hinchey EJ, Schaal PG, Richards GK (1978). Treatment of perforated diverticular disease of the colon. Adv Surg.

[7] Kaiser AM, Jiang JK, Lake JP (2005). The management of complicated diverticulitis and the role of computed tomography. Am J Gastroenterol.

[8] Galetin T, Galetin A, Vestweber KH, Rink AD (2018). Systematic review and comparison of national and international guidelines on diverticular disease. Int JColorectal Dis.

[9] Schultz JK, Azhar N, Binda GA (2020). European Society of Coloproctology: guidelines for the management of diverticular disease of the colon. Colorectal Dis..

[10] Ross JT, Matthay MA, Harris HW (2018). Secondary peritonitis: principles of diagnosis and intervention. BMJ.

[11] Andeweg CS, Mulder IM, Felt-Bersma RJ (2013). Guidelines of diagnostics and treatment of acute left-sided colonic diverticulitis. Dig Surg.

[12] Kiewiet JJ, Andeweg CS, Laurell H (2014). External validation of two tools for the clinical diagnosis of acute diverticulitis without imaging. Dig Liver Dis.

[13] Ambrosetti P (2016). Acute left-sided colonic diverticulitis: clinical expressions, therapeutic insights, and role of computed tomography. Clin Exp Gastroenterol.

[14] Ritz JP, Lehmann KS, Loddenkemper C, Frericks B, Buhr HJ, Holmer C (2010). Preoperative CT staging in sigmoid diverticulitis does it correlate with intraoperative and histological findings?. Langenbecks Arch Surg.

[15] Gielens MPM, Mulder IM, van der Harst E (2012). Preoperative staging of perforated diverticulitis by computed tomography scanning. Tech Coloproctol.

[16] Lohrmann C, Ghanem N, Pache G (2005). CT in acute perforated sigmoid diverticulitis. Eur J Radiol.

[17] Zago M, Mariani D, Casamassima A (2016). Impact of systematic use of US-guided DPA in the management of abdominal emergencies. Eur J Trauma Emerg Surg.

[18] van Dijk ST, Doelare SAN, van Geloven AAW, Boermeester MA (2018). A systematic review of pericolic extraluminal air in left-sided acute colonic diverticulitis. Surg Infect (Larchmt).

[19] Sallinen VJ, Mentula PJ, Leppäniemi AK (2014). Nonoperative management of perforated diverticulitis with extraluminal air is safe and effective in selected patients. Dis Colon Rectum.

[20] Titos-García A, Aranda-Narváez JM, Romacho-López L, Gonzales-Sanchez AJ, Cabrera-Serna I, Santoyo-Santoyo J (2017). Nonoperative management of perforated acute diverticulitis with extraluminal air: results and risk factors of failure. Int J Colorectal Dis.

[21] Thorisson A, Nikberg M, Andreasson K, Smedh K, Chabok A (2018). Non-operative management of perforated diverticulitis with extraluminal or free air—a retrospective single center cohort study. Scand J Gastroenterol.

[22] Dharmarajan S, Hunt SR, Birnbaum EH, Fleshman JW, Mutch MG (2011). The efficacy of nonoperative management of acute complicated diverticulitis. Dis Colon Rectum.

[23] Colas PA, Duchalais E, Duplay Q (2017). Failure of conservative treatment of acute diverticulitis with extradigestive air. World J Surg.

[24] Singer M, Deutschman CS, Seymour CW (2016). The third international consensus definitions for sepsis and septic shock (Sepsis-3). JAMA.

[25] Tridente A, Clarke GM, Walden A (2014). Patients with faecal peritonitis admitted to European intensive care units: an epidemiological survey of the GenOSept cohort. Intensive Care Med.

[26] Sartelli M, Abu-Zidan FM, Labricciosa FM (2019). Physiological parameters for Prognosis in Abdominal Sepsis (PIPAS) Study: a WSES observational study. World J Emerg Surg.

[27] Hansen O, Graupe F, Stock W (1998). Prognostic factors in perforating diverticulitis of the large intestine. Chirurg.

[28] Biondo S, Ramos E, Deiros M (2000). Prognostic factors for mortality in left colonic peritonitis: a new scoring system. J Am Coll Surg.

[29] Sallinen VJ, Leppäniemi AK, Mentula PJ (2015). Staging of acute diverticulitis based on clinical, radiologic, and physiologic parameters. J Trauma Acute Care Surg.

[30] ATLS Committee (2018) Shock. In: ATLS advanced trauma life support. student course manual, 10th edn. American College of Surgeons, Chicago, pp 42–61.

[31] Greilsamer T, Abet E, Meurette G (2017). Is the failure of laparoscopic peritoneal lavage predictable in Hinchey III diverticulitis management?. Dis Colon Rectum.

[32] Binda GA, Bonino MA, Siri G (2018). Multicentre international trial of laparoscopic lavage for Hinchey III acute diverticulitis (LLO Study). Br J Surg.

[33] Vermeulen J, Gosselink MP, Hop WC (2011). Long-term survival after perforated diverticulitis. Colorectal Dis.

[34] Ince M, Stocchi L, Khomvilai S, Kwon DS, Hammel JP, Kiran MP (2012). Morbidity and mortality of the Hartmann procedure for diverticular disease over 18 years in a single institution. Colorectal Dis.

[35] Golda T, Kreisler E, Mercader C (2014). Emergency surgery for perforated diverticulitis in the immunosuppressed patient. Colorectal Dis.

[36] Nandan AR, Bohnen JD, Sangji NF (2017). The Emergency Surgery Score (ESS) accurately predicts the occurrence of postoperative complications in emergency surgery patients. J Trauma Acute Care Surg.

[37] Bertsimas D, Dunn J, Velmahos GC, Kaafaarani HMA (2018). Surgical risk is not linear: derivation and validation of a novel, user-friendly, and machine-learning-based predictive optimal trees in emergency surgery risk (POTTER) calculator. Ann Surg.

[38] Sartelli M, Abu-Zidan FM, Catena F (2015). Global validation of the WSES Sepsis Severity Score for patients with complicated intra-abdominal infections: a prospective multicentre study (WISS Study). World J Emerg Surg.

[39] Linder MM, Wacha H, Feldmann U, Wesch H, Streifensand RA, Gundlach E (1987). The Mannheim peritonitis index. An instrument for the intraoperative prognosis of peritonitis. Chirurg.

[40] Tolonen M, Sallinen V, Leppäniemi A, Backlund M, Mentula P (2019). The role of the intra-abdominal view in complicated intra-abdominal infections. World J Emerg Surg.

[41] Biondo S, Ramos E, Fraccalvieri D, Kreisler E, Raguè JM, Jaurrieta E (2006). Comparative study of left colonic Peritonitis Severity Score and Mannheim Peritonitis Index. Br J Surg.

[42] Cirocchi R, Fearnhead N, Vettoretto N (2019). The role of emergency laparoscopic colectomy for complicated sigmoid diverticulits: a systematic review and meta-analysis. Surgeon.

[43] Dreifuss NH, Schlottmann F, Piatti JM, Bun ME, Rotholtz NA (2020). Safety and feasibility of laparoscopic sigmoid resection without diversion in perforated diverticulitis. Surg Endosc.

[44] Bemelman WA (2018). Perforated sigmoid diverticulitis: Hartmann’s procedure or resection with primary anastomosis. Tech Coloproctol.

[45] O'Sullivan GC, Murphy D, O'Brien MG (1996). Laparoscopic management of generalized peritonitis due to perforated colonic diverticula. Am J Surg.

[46] Taylor CJ, Layani L, Ghusn MA (2006). Perforated diverticulitis managed by laparoscopic lavage. ANZ J Surg.

[47] Myers E, Hurley M, O’Sullivan GC (2008). Laparoscopic peritoneal lavage for generalized peritonitis due to perforated diverticulitis. Br J Surg.

[48] Sorrentino M, Brizzolari M, Scarpa E (2015). Laparoscopic peritoneal lavage for perforated colonic diverticulitis: a definitive treatment? Retrospective analysis of 63 cases. TechColoproctol.

[49] Toorenvliet BR, Swank H, Schoones JW, Hamming JF, Bemelman WA (2009). Laparoscopic peritoneal lavage for perforated colonic diverticulitis: a systematic review. Colorectal Dis.

[50] Schultz JK, Yaqub S, Wallon C (2015). Laparoscopic lavage vs primary resection for acute perforated diverticulitis: the SCANDIV Randomized Clinical Trial. JAMA.

[51] Vennix S, Musters GD, Mulder IM (2015). Laparoscopic peritoneal lavage or sigmoidectomy for perforated diverticulitis with purulent peritonitis: a multicentre, parallel-group, randomised, open-label trial. Lancet.

[52] Thornell A, Angenete E, Bisgaard T (2016). Laparoscopic lavage for perforated diverticulitis with purulent peritonitis: a randomized trial. Ann Intern Med.

[53] Cirocchi R, Di Saverio S, Weber DG (2017). Laparoscopic lavage versus surgical resection for acute diverticulitis with generalised peritonitis: a systematic review and meta-analysis. Tech Coloproctol.

[54] Galbraith N, Carter JV, Netz U (2017). Laparoscopic lavage in the management of perforated diverticulitis: a contemporary meta-analysis. J Gastrointest Surg.

[55] Marshall JR, Buchwald PL, Gandhi J (2016). Laparoscopic lavage in the management of Hinchey Grade III diverticulitis: a systematic review. Ann Surg.

[56] Penna M, Markar SR, Mackenzie H (2017). Laparoscopic lavage versus primary resection for acute perforated diverticulitis: review and meta-analysis. Ann Surg.

[57] Shaikh FM, Stewart PM, Walsh SR (2017). Laparoscopic peritoneal lavage or surgical resection for acute perforated sigmoid diverticulitis: a systematic review and meta-analysis. Int J Surg.

[58] Acuna SA, Wood T, Chesney TR (2018). Operative strategies for perforated diverticulitis: a systematic review and meta-analysis. Dis Colon Rectum.

[59] Schmidt S, Ismail T, Puhan MA (2018). Meta-analysis of surgical strategies in perforated left colonic diverticulitis with generalized peritonitis. Langenbecks Arch Surg.

[60] Slim K, Le Roy B (2017). Laparoscopic peritoneal lavage for perforated sigmoid diverticulitis—an example of surgical research failure. Colorectal Dis.

[61] Gehrman J, Angenete E, Bjorholt I (2016). Health economic analysis of laparoscopic lavage versus Hartmann's procedure for diverticulitis in the randomized DILALA trial. Br J Surg.

[62] Angenete E, Bock D, Rosenberg J, Haglind E (2017). Laparoscopic lavage is superior to colon resection for perforated purulent diverticulitis—a meta-analysis. Int J Colorectal Dis.

[63] Vennix S, van Dieren S, Opmeer BC, Lange JF, Bemelman WA (2017). Cost analysis of laparoscopic lavage compared with sigmoid resection for perforated diverticulitis in the Ladies trial. Br J Surg.

[64] Sneiders D, Lambrichts DPV, Swank HA (2019). Long-term follow-up of a multicentre cohort study on laparoscopic peritoneal lavage for perforated diverticulitis. Colorectal Dis.

[65] Kohl A, Rosenberg J, Bock D (2018). Two-year results of the randomized clinical trial DILALA comparing laparoscopic lavage with resection as treatment for perforated diverticulitis. Br J Surg.

[66] Binda GA, Karas JR, Serventi A (2012). Primary anastomosis vs nonrestorative resection for perforated diverticulitis with peritonitis: a prematurely terminated randomized controlled trial. Colorectal Dis.

[67] Oberkofler CE, Rickenbacher A, Raptis DA (2012). A multicenter randomized clinical trial of primary anastomosis or Hartmann's procedure for perforated left colonic diverticulitis with purulent or fecal peritonitis. Ann Surg.

[68] Bridoux V, Regimbeau JM, Ouaissi M (2017). Hartmann's procedure or primary anastomosis for generalized peritonitis due to perforated diverticulitis: a prospective multicenter randomized trial (DIVERTI). J Am Coll Surg.

[69] Schmidt S, Ismail T, Puhan MA, Soll C, Breitenstein S (2018). Meta-analysis of surgical strategies in perforated left colonic diverticulitis with generalized peritonitis. Langenbecks Arch Surg.

[70] Cirocchi R, Afshar S, Shaban F (2018). Perforated sigmoid diverticulitis: Hartmann's procedure or resection with primary anastomosis-a systematic review and meta-analysis of randomised control trials. Tech Coloproc.

[71] Lambrichts DPV, Edomskis PP, van der Bogt RD, Kleinrensink GJ, Bemelman WA, Lange JF (2020). Sigmoid resection with primary anastomosis versus the Hartmann’s procedure for perforated diverticulitis with purulent or fecal peritonitis: a systematic review and meta-analysis. Int J Colorectal Dis.

[72] Lambrichts DPV, Vennix S, Musters GD (2019). Hartmann's procedure versus sigmoidectomy with primary anastomosis for perforated diverticulitis with purulent or faecal peritonitis (LADIES): a multicentre, parallel-group, randomised, open-label, superiority trial. Lancet Gastroenterol Hepatol.

[73] Gachabayov M, Oberkofler CE, Tuech JJ, Hahnloser D, Bergamaschi R (2018). Resection with primary anastomosis vs non restorative resection for perforated diverticulitis with peritonitis: a systematic review and meta-analysis. Colorectal Dis.

[74] Etzioni DA, Mack TM, Beart RW (2009). Diverticulitis in the United States: 1998–2005: changing patterns of disease and treatment. Ann Surg.

[75] Issa N, Dreznik Z, Dueck DS (2009). Emergency surgery for complicated acute diverticulitis. Colorectal Dis.

[76] Tabbara M, Velmahos GC, Butt MU (2010). Missed opportunities for primary repair in complicated acute diverticulitis. Surgery.

[77] Morris CR, Harvey IM, Stebbings WS, Hart AR (2008). Incidence of perforated diverticulitis and risk factors for death in a UK population. Br J Surg.

[78] Wong NY, Eu KW (2005). A defunctioning ileostomy does not prevent clinical anastomotic leak after a low anterior resection: a prospective, comparative study. Dis Colon Rectum.

[79] Regenet N, Tuech JJ, Pessaux P (2002). Intraoperative colonic lavage with primary anastomosis vs. Hartmann's procedure for perforated diverticular disease of the colon: a consecutive study. Hepatogastroenterology.

[80] Biondo S, Jaurrieta E, Jorba R (1997). Intraoperative colonic lavage and primary anastomosis in peritonitis and obstruction. Br J Surg.

[81] Ortiz H, Biondo S, Ciga MA (2009). Comparative study to determine the need for intraoperative colonic irrigation for primary anastomosis in left-sided colonic emergencies. Colorectal Dis.

[82] Moore FA, Moore EE, Burlew CC (2012). Western Trauma Association critical decisions in trauma: management of complicated diverticulitis. J Trauma Acute Care Surg.

[83] Diaconescu B, Uranues S, Fingerhut A (2020). The Bucharest ESTES consensus statement on peritonitis. Eur J Trauma Emerg Surg.

[84] Lamontagne F, Richards-Belle A, Thomas K (2020). Effect of Reduced Exposure to Vasopressors on 90-Day Mortality in Older Critically Ill PatientsWith Vasodilatory Hypotension: A Randomized Clinical Trial. JAMA.

[85] Maheshwari K, Nathanson BH, Munson SH (2018). The relationship between ICU hypotension and in-hospital mortality and morbidity in septic patients. Intensive Care Med.

[86] Shani V, Muchtar E, Kariv G (2010). Systematic review and meta-analysis of the efficacy of appropriate empiric antibiotic therapy for sepsis. Antimicrob Agents Chemother.

[87] Solomkin JS, Mazuski JE, Bradley JS (2010). Diagnosis and management of complicated intra-abdominal infection in adults and children: guidelines by the Surgical Infection Society and the Infectious Diseases Society of America. Clin Infect Dis.

[88] Pea F, Viale P (2009). Bench-to-bedside review: appropriate antibiotic therapy in severe sepsis and septic shock–does the dose matter?. Crit Care.

[89] Wong PF, Gilliam AD, Kumar S, Shenfine J, O’Dair GN, Leaper DJ (2005). Antibiotic regimens for secondary peritonitis of gastrointestinal origin in adults. Cochrane Database Syst Rev..

[90] Sartelli M, Weber DG, Ruppé E (2016). Antimicrobials: a global alliance for optimizing their rational use in intra-abdominal infections (AGORA). World J Emerg Surg.

[91] Dadgostar P (2019). Antimicrobial Resistance: Implications and Costs. Infect Drug Resist.

[92] Tan WJ, Ng WQ, Sultana R (2018). Systematic review and meta-analysis of the use of serum procalcitonin levels to predict intra-abdominal infections after colorectal surgery. Int J Colorectal Dis.

[93] Sager R, Kutz A, Mueller B (2017). Procalcitonin-guided diagnosis and antibiotic stewardship revisited. BMC Med.

[94] Gustafsson UO, Scott MJ, Hubner M (2019). Guidelines for perioperative care in elective colorectal surgery: enhanced recovery after surgery (ERAS) society recommendations: 2018. World J Surg.

[95] Beverly A, Kaye AD, Ljungqvist O, Urman RD (2017). Essential elements of multimodal analgesia in enhanced recovery after surgery (ERAS) guidelines. Anesthesiol Clin.

[96] Chou R, Gordon DB, de Leon-Casasola OA (2016). Management of postoperative pain: a clinical practice guideline from the American Pain Society, the American Society of Regional Anesthesia and Pain Medicine, and the American Society of Anesthesiologists' Committee on Regional Anesthesia, Executive Committee, and Administrative Council. J Pain.

[97] Maund E, McDaid C, Rice S, Wright K, Jenkins B, Woolacott N (2011). Paracetamol and selective and non-selective non-steroidal anti-inflammatory drugs for the reduction in morphine-related side-effects after major surgery: a systematic review. Br J Anaesth.

[98] Peng F, Liu S, Hu Y (2016). Influence of perioperative nonsteroidal anti-inflammatory drugs on complications after gastrointestinal surgery: A meta-analysis. Acta Anaesthesiol Taiwan.

[99] Huang Y, Tang SR, Young CJ (2018). Nonsteroidal anti-inflammatory drugs and anastomotic dehiscence after colorectal surgery: a meta-analysis. ANZ J Surg.

[100] Modasi A, Pace D, Godwin M, Smith C, Curtis B (2019). NSAID administration post colorectal surgery increases anastomotic leak rate: systematic review/meta-analysis. Surg Endosc.

[101] Jamjittrong S, Matsuda A, Matsumoto S (2019). Postoperative non-steroidal anti-inflammatory drugs and anastomotic leakage after gastrointestinal anastomoses: systematic review and meta-analysis. Ann Gastroenterol Surg.

[102] Guay J, Nishimori M, Kopp SL (2016). Epidural local anesthetics versus opioid-based analgesic regimens for postoperative gastrointestinal paralysis, vomiting, and pain after abdominal surgery: a cochrane review. Anesth Analg.

[103] El-Boghdadly K, Madjdpour C, Chin KJ (2016). Thoracic paravertebral blocks in abdominal surgery—a systematic review of randomized controlled trials. Br J Anaesth.

[104] Vather R, Trivedi S, Bissett I (2013). Defining postoperative ileus: results of a systematic review and global survey. J Gastrointest Surg.

[105] Boeckxstaens GE, de Jonge WJ (2009). Neuroimmune mechanisms in postoperative ileus. Gut.

[106] Sternini C, Patierno S, Selmer IS (2004). The opioid system in the gastrointestinal tract. Neurogastroenterol Motil.

[107] Venara A, Meillat H, Cotte E (2020). Incidence and risk factors for severity of postoperative ileus after colorectal surgery: a prospective registry data analysis. World J Surg.

[108] Alhashemi M, Fiore JF, Safa N (2019). Incidence and predictors of prolonged postoperative ileus after colorectal surgery in the context of an enhanced recovery pathway. Surg Endosc.

[109] Bragg D, El-Sharkawy AM, Psaltis E, Psaltis E, Maxwell-Armstrong CA, Lobo DN (2015). Postoperative ileus: recent developments in pathophysiology and management. Clin Nutr.

[110] Stakenborg N, Labeeuw E, Gomez-Pinilla PJ (2019). Preoperative administration of the 5-HT4 receptor agonist prucalopride reduces intestinal inflammation and shortens postoperative ileus via cholinergic enteric neurons. Gut.

[111] Roslan F, Kushairi A, Cappuyns L (2020). The impact of sham feeding with chewing gum on postoperative ileus following colorectal surgery: a meta-analysis of randomised controlled trials. J Gastrointest Surg..

[112] Drake TM, Ward AE (2016). Pharmacological management to prevent ileus in major abdominal surgery: a systematic review and meta-analysis. J Gastrointest Surg.

[113] Vaughan-Shaw PG, Fecher IC, Harris S (2012). A meta-analysis of the effectiveness of the opioid receptor antagonist alvimopan in reducing hospital length of stay and time to GI recovery in patients enrolled in a standardized accelerated recovery program after abdominal surgery. Dis Colon Rectum.

[114] Xu LL, Zhou XQ, Yi PS (2016). Alvimopan combined with enhanced recovery strategy for managing postoperative ileus after open abdominal surgery: a systematic review and meta-analysis. J Surg Res.

[115] Touchette DR, Yang Y, Tiryaki F (2012). Economic analysis of alvimopan for prevention and management of postoperative ileus. Pharmacotherapy.

[116] Binda GA, Serventi A, Puntoni M, Amato A (2015). Primary anastomosis versus Hartmann's procedure for perforated diverticulitis with peritonitis: an impracticable trial. Ann Surg.

[117] Baldock TE, Brown LR, McLean RC (2019). Perforated diverticulitis in the North of England: trends in patient outcomes, management approach and the influence of subspecialisation. Ann R Coll Surg Engl.

[118] Goldstone RN, Cauley CE, Chang DC, Kunitake H, Ricciardi R, Bordeianou L (2019). The effect of surgical training and operative approach on outcomes in acute diverticulitis: should guidelines be revised?. Dis Colon Rectum.

[119] Lee JM, Bai P, Chang J (2019). Hartmann's procedure vs primary anastomosis with diverting loop ileostomy for acute diverticulitis: nationwide analysis of 2,729 emergency surgery patients. J Am Coll Surg..

[120] Boyce S, Bartolo D, Paterson H (2013). Subspecialist emergency management of diverticulitis is associated with reduced mortality and fewer stomas. Colorectal Dis.

[121] Lohsiriwat V, Jitmungngan R (2019). Enhanced recovery after surgery in emergency colorectal surgery. Review of literature and current practices. World J Gastrointest Surg.

